# Molecular characterization of the genome-wide BOR transporter gene family and genetic analysis of *BnaC04.BOR1;1c* in *Brassica napus*

**DOI:** 10.1186/s12870-018-1407-1

**Published:** 2018-09-14

**Authors:** Haifei Chen, Quan Zhang, Mingliang He, Sheliang Wang, Lei Shi, Fangsen Xu

**Affiliations:** National Key Laboratory of Crop Genetic Improvement, Microelement Research Center, Huazhong Agricultural University, Wuhan, 430070 China

**Keywords:** *Brassica napus*, BOR transporter, B deficiency, Transcriptional profile, B efficiency, Gene function

## Abstract

**Background:**

Boron (B) deficiency is an agricultural problem that causes significant losses of crop yield in many areas of the world. However, systematic analysis of BOR family genes for B transport in rapeseed is still lacking. We aimed to identify and characterize BOR transporters in *Brassica napus* and the potential role of these transporters in B homeostasis in response to B deficiency.

**Results:**

Here, we identified 20 BOR transporters from the *Brassica napus* genome, which were classified into six distinct groups that represent clear orthologous relationships to their family members in *Arabidopsis*. qRT-PCR revealed distinct expression profiles for *BnBOR*s in different tissues and in response to external B levels. The B-efficient cultivar QY10 accumulated more B in shoots than the B-inefficient cultivar W10, and overexpression of *BnaBOR1;1c* could alleviate shoot B-deficiency symptoms in W10 by distributing more B from roots to shoots. Additionally, *BnBOR1;1c* expression was up-regulated by B deficiency, and the induction of *BnBOR1;1c* was more intense in QY10. Moreover, two conserved InDels were found in the promoter regions of *BnBOR1;1c* within different B-efficient genotypes.

**Conclusions:**

Overall, the molecular characterization of the *BnBOR* genes of two B-efficient cultivars and their responses to B deficiency highlights the diversity of the family members in *B*. *napus*, and *BnaC4.BOR1;1c* has potential as a candidate gene for improving B nutrition.

**Electronic supplementary material:**

The online version of this article (10.1186/s12870-018-1407-1) contains supplementary material, which is available to authorized users.

## Background

Boron (B) is an essential micronutrient for higher plants [1]. In terms of physiological functions, B is important for cell wall (CW) structure, as it crosslinks the pectic polysaccharide rhamnogalacturonan II (RG-II) and pectin assembly in the CW [2, 3]. Because of the difficulty transferring B in plants, B deficiency primarily restrains rapidly growing tissues, inhibiting apical growth in the roots and shoots, and reduces fertility [4–6]. Most importantly, B deficiency often causes dried-up floral buds, fewer pods and low seed yield or sterility in plant reproductive growth.

Among essential mineral nutrients, B has the narrowest margin in soil concentrations between deficiency and toxicity [7]. A number of genes involved in efficient B uptake, transport, partitioning and export have been identified as necessary for tolerance to B deficiency and toxicity [8]. In *Arabidopsis*, AtBOR1 has been identified as an efflux type transporter for xylem loading and is essential for preventing shoots from B deficiency [9, 10]. Under B-deficient conditions, although *AtBOR1* mRNA accumulation is not abundant, its protein showed high abundance in the PM [11]. Furthermore, BOR1 activity is repressed through ubiquitination-mediated vacuolar trafficking to avoid B toxicity under high B conditions [12, 13]. Subsequently, homologues of AtBOR1 are assumed to play crucial roles in the resistance to B deprivation in rice (OsBOR1) [14], grapevine (*Vitis vinifera* L.) (VvBOR1) [15], *Citrus macrophylla* (CmBOR1) [16], wheat (*Triticum awstivum* L.) (TaBOR1) [17], tomato [18] and *B. napus* (BnBOR1;1c) [19]. OsBOR1 is required for B uptake and xylem loading under B deficiency conditions [14]. Recently, the B exporter BOR2 was found to differ from BOR1 in *Arabidopsis*; it is required for the effective crosslinking of the pectin polysaccharide RG-II and root cell elongation under B limitation [20]. Additionally, AtBOR4, an *Arabidopsis* borate efflux transporter, is significant for the directional export of B from roots to soils to prevent the overaccumulation of B in the xylem and improve the tolerance to excess B [21]. Bot1 contributes to the high B tolerance of Sahara, a barley landrace, by exclusion of B from the roots [22]. To date, the boric acid/borate transporter activity of BORs in monocotyledon and dicotyledon plants have been demonstrated and identified; these plants have a conserved sorting motif and the boric acid channel showed homology to the mammalian Slc4 bicarbonate (HCO_3_^−^) family [23, 24].

Allotetraploid rapeseed (*Brassica napus* L., AnAnCnCn, 2n = 38, 840 Mb), which originated from a natural hybridization between *Brassica rapa* (ArAr, 2n = 20, 312 Mb) and *Brassica oleracea* (CoCo, 2n = 18, 540 Mb) approximately 7500–12,500 years ago, is extremely sensitive to B deficiency and shows a notable reduction in seed yield and quality with a low B supply [25–27]. In recent years, because borate rock is a depletable and non-renewable mineral resource, numerous effective measures have been taken to address this problem, including the application of borate fertilizers to soils with low B abundance. A molecular understanding of B deficiency responses in plants is pivotal for developing crop varieties with high B use efficiency under low B conditions. Such understanding is rapidly progressing for BOR1 function in *Brassica napus*. Six B transporter genes (*BnBOR1s*) homologous to *AtBOR1* were identified and divided into three groups in *B. napus*. Each group was comprised of two members, one of which originated from *B. rapa* (*BnBOR1;1a*, *BnBOR1;2a*, and *BnBOR1;3a*) and the other from *B. oleracea* (*BnBOR1;1c*, *BnBOR1;2c*, and *BnBOR1;3c*) [28]. Unlike the non-transcriptional regulation of *AtBOR1*, *BnaC4.BOR1;1c* (*BnBOR1;1c*) is highly expressed not only in roots but also in shoot nodal regions and flowers in response to B limitation, and is critical for the development and fertility of inflorescences in rapeseed [19].

Here, we report the systematic analysis of the gene structure, phylogeny, motif composition, chromosomal localization and expression patterns of BOR genes in *B. napus* under different B conditions. Furthermore, transgenic *BnaC4.BOR1;1c* knockdown and overexpression lines in the *B. napus* cultivars QY10 and Westar 10 were established as examples to study the functional relationship between B transporters and B efficiency.

## Methods

### Identification and physicochemical parameter analysis of *BOR* genes in *B. napus*

All BOR genes were identified in *B. napus* based on their homology similarity to the 7 BOR protein sequences in *Arabidopsis* from the TAIR10 database (http://www.arabidopsis.org/index.jsp) using the BLAST search program in the CNS-Genoscope database (http://www.genoscope.cns.fr/brassicanapus/) [27]. The exon-intron structures of the BnBOR family members were investigated based on coding sequence alignments with corresponding genomic sequences, and the diagram was drawn using the online Gene Structure Display Server (GSDS; http://gsds.cbi.pku.edu.cn/) [28]. The physicochemical parameters, including molecular weight (MW) and isoelectronic point (pI), for each BnBOR protein were calculated using the compute pI/MW tool in ExPASy (http://www.expasy.org/tools/). GRAVY (grand average of hydropathicity) values were calculated using the PROTPARAM tool (http://web.expasy.org/protparam/).

### Chromosome localization

To determine the physical locations of the *BnBOR* genes, the starting and ending positions of all *BnBOR* genes on each chromosome were obtained from the *Brassica* database (BRAD; http://www.genoscope.cns.fr/brassicanapus/). The MapInspect software was used to draw the gene chromosome location diagrams. The 20 *BnBOR* gene members were distributed non-randomly on 12 *B. napus* chromosomes between chromosomes A and C. Chromosome A03 and C04 contained the most *BnBOR* genes with three, whereas chromosomes A02, A05, A06, C01, C02 and C05 contained one gene each.

### *BnBOR* member gene structure and conserved motif analysis

Conserved motif structures encoded by the *BnBOR* family genes were identified by the Multiple Expectation Maximization for Motif Elicitation (MEME) program version 4.11.2 (http://meme-suite.org/tools/meme) [29]. The parameter settings included output motifs (10), minimum motif width (10), and maximum motif width (100). The MEME motifs were annotated using the Pfam (http://pfam.xfam.org/search) and NCBI databases.

### Phylogenetic analysis

Multiple sequence alignment of all the predicted BOR genes from *B. napus* and *Arabidopsis* was performed using the NCBI BLASTP (Target type: Proteome) and ClustalW2 programs with default parameters. The 37 BOR amino acid sequences (Supplemental data for the amino acids) from two taxonomic families containing core conserved BORs were downloaded using the Phytozome 11 online software from the Joint Genome Institute. These 37 BOR amino acid sequences were from four plant species including *Brassica napus*, *Arabidopsis*, *Oryza sativa* and *Zea mays*. The evolutionary history was inferred using the Neighbour-Joining method [30], and the numbers displayed in the phylogenetic tree represent bootstrap values that were estimated (with 500 replicates) to assess the relative support for each branch using MEGA6.0 software.

### Plant materials and treatments

Plump seeds from the B-efficient cultivar QY10, Ningyou 7 and B-inefficient cultivar W10, Bakow were used for hydroponic culture experiments in an illuminated growth room at 24/22 °C (day/night) under a photoperiod 14/10 h (light/dark) with a light density of 300–320 μmol m^− 2^ s^− 1^. The samples were surface-sterilized for 15 min using 0.5% NaClO (*w*/*v*) and rinsed completely with sterilized ultrapure water (> 18.25 MΩ·cm). The seeds were then sown on moistened gauze after being soaked in deionized water for 1 d. After 5 d of germination, the uniform seedlings were transplanted into 10-L black plastic containers filled with Hoagland and Arnon solution with 0.25 μM B (low B) and 25 μM B (high B) for 7 d or 10 d. The nutrient solution was replaced every 3 days. The rapeseed seedlings were first grown in one quarter-strength solution, afterwards progressing to one-half-strength and eventually full-strength.

### RNA extraction, reverse transcription and real-time quantitative PCR

Total RNA was extracted from plant fresh samples independently using the RNAiso™ Plus reagent (Takara Bio, Otsu, Shiga, Japan) according to the manufacturer’s recommendations. RNA samples were treated with RNase-free DNase I (Invitrogen, Grand Island, NY, USA). Subsequently, first strand cDNA was synthesized using the PrimeScript™ RT Master Mix (Takara, Tokyo, Japan) according to the manufacturer’s protocol. The specific primers for the BnBOR genes were designed with Primer-NCBI (http://www.ncbi.nlm.nih.gov/tools/primersblast/index.cgi?LINK=BlastHome) and are listed in Additional file 1: Table S1. Real-time fluorescence quantitative PCR (RT-qPCR) to detect the expression of the target genes was performed on a CFX96™ Real-Time PCR Detection System (Bio-Rad, Hercules, CA, USA) with the SYBR Green Real-Time PCR Master Mix Kit (TOYOBO, Japan). The PCR conditions were as follows: 95 °C for 5 min, followed by 40 cycles of 95 °C for 10 s, 60 °C for 15 s and 72 °C for 20 s. The reference gene *BnaActin* was used as an internal control, and the fold change was analysed via the 2^-ΔΔCt^ method [31]. The gene-specific primers are listed in Additional file 1: Table S1. The primer specificity and PCR products were confirmed by sequencing and checked on the NCBI website (https://www.ncbi.nlm.nih.gov/) or rapeseed database using Blast (Http://www.genoscope.cns.fr/brassicanapus). The primer efficiency was checked according to Zhang et al. [19]

### Characterization of *cis*-elements in the *BnaC4.BOR1;1c* promoter region in *B. napus*

The 2000-bp upstream sequence relative to the translation start codon in *BnaC4.BOR1;1c* was downloaded from CNS-Genoscope. The *BnaC4.BOR1;1c* promoter was analysed to determine the *cis*-regulatory elements using the plant *cis*-element database PlantCARE [32].

### Vector construction and plant transformation

To obtain the *BnaC4.BOR1;1c* overexpression (OE) lines, a full-length *BnaC4.BOR1;1c* gene coding sequence was amplified with *Pfu* DNA polymerase (Promega, Madison, WT) using gene-specific primers (Additional file 1: Table S1). The amplicon was ligated into the pGEMT easy vector (Promega, Madison, WI) and confirmed by DNA sequencing. Then, the coding sequence was subsequently cloned into the XbaІ and XhoІ sites in the pBinGlyRed3 vector with the 2 × 35S promoter using DNA fusion technology. The vector was introduced into the *Agrobacterium tumefaciens* strain GV3101 by electroporation. W10 were transformed according to the method in [33]. Putative transformants (T0) were transferred to soil for growth. The genomic DNA was isolated from young leaves and used to determine the positive plants by PCR using vector-specific primers. Seedlings from the T1 generation were examined for 3:1 segregation, and the seedlings with an OE construction (either heterozygous or homozygous) were kept to obtain the T2 generation. Expression of *BnaC4.BOR1;1c* in homozygous OE lines was determined by quantitative RT-PCR to detect the efficiency using specific primer pairs. Three homozygous lines were selected for further analysis. The QY10, W10 and *BnaC4.BOR1;1c* OE lines were grown hydroponically for 10 d under normal (25 μM) and low (0.25 μM) B stress as described above.

### Transmission electron microscopy

For transmission electron microscopy (TEM) analysis, juvenile leaves (approximately 1 mm^2^) from the fresh seedlings were sampled and immediately fixed in 2.5% glutaraldehyde in 0.1 M sodium cacodylate buffer, pH 7.4, containing 2% sucrose for 2 h. Post-fixation was performed in 1% osmium tetroxide in the same buffer for 1.5 h. The samples were then dehydrated using an ethanol series (30%, 50%, 70%, 80%, 90%, 95% and 100% [*v*/*v*] ethanol) and propylene oxide for dehydration and embedded in epoxy resin. Ultra-thin 0.5–1.0 μm sections were cut with an ultramicrotome (Leica UC6/FC6, Germany), and a transmission electron microscope (HITACHI, H7650, Japan) was used to examine uranyl acetate-stained sections at 40 kV–120 kV.

### Boron measurement

The samples (root and shoot) were separated and dried at 105 °C for 30 min and then to a constant weight at 65 °C. The dried samples were ground into fine powders using a carnelian mortar, and B was lixiviated with 10 mL of 1 M HCl on a 250-rpm shaker for 2 h. The B concentration was measured using an inductively coupled plasma mass spectrometer (ICP-MS; Perkin Elmer, ELAN DRC-e, USA). B accumulation was calculated as the B concentration × dry weight.

### Statistical analysis

Each graphical plot represents the results from multiple independent experiments (*n* ≥ 3), and the values are the means ± SD. Statistical significance between two genotypes or two organs in the same B condition was used independent Student’s t-test. LSD test was used for multiple comparisons at the *p* < 0.05 level among the different B treatments and genotypes in the same organ. Data were analyzed in SPSS 18.0 software and *p* value < 0.05 was considered statistically significant.

## Results

### Genome-wide identification of *BnBOR* gene family members in *B. napus*

A total of 20 *BOR* genes (*BnBOR1s*-*BnBOR7s*) were identified in the *B. napus* genome based on their homology to the 7 BOR protein sequences in *Arabidopsis* from the TAIR10 database using the BLAST search program in the CNS-Genoscope database*.* The systematic analysis of the BnBORs is shown in Table 1. Large variations in the encoding amino acid (aa) length of these genes were found, with lengths ranging from 660 aa (BnBOR7A03) to 738 aa (BnBOR3A03), and the molecular weights range from 74.67 to 81.24 kDa. ExPASy analysis revealed that these protein sequences have stable isoelectric points (pI) ranging from 6.91 to 9.26, and most of the sequence have similar parameters. Almost all BOR proteins have relatively high isoelectric points (pI > 7), except for BnBOR4A02 and BnBOR4C05. GRAVY values are defined as the sum of the hydropathy values of all amino acids divided by the protein length. All of the BnBORs are hydrophilic, with values ranging from 0.162 to 0.266. Additionally, TargetP and WoLF PSORT were used to predict the subcellular location of the 20 BnBOR proteins, which were similar to BnaC4.BOR1;1c located in the cell PM, implying the functions of the *BnBOR* family members. These results for the parameter analysis indicate that *BnBOR* family members have stable essential characteristics, physicochemical properties and an abundant chromosomal distribution.

**Table 1:** Gene sequence characteristics of 20 BnBORs and their protein physicochemical parameters

BnaBORs	Mapping	ID	Name	Description					Physicochemical parameters	
				Length (bp)	Size (Aa)	Intron	Exon	Weight (kDa)	pI	GRAVY
BnaBOR1s	A04	GSBRNA2T00003348001	BnaA04g26910D	2106	701	9	10	78.24	8.68	0.195
	C04	GSBRNA2T00038178001	BnaC04g51480D	2106	701	9	10	78.31	8.77	0.183
	A05	GSBRNA2T00132977001	BnaA05g00740D	2115	704	9	10	78.6	8.86	0.194
	C04	GSBRNA2T00064725001	BnaC04g00350D	2115	704	9	10	78.64	8.86	0.200
	A03	GSBRNA2T00138903001	BnaA03g21650D	2112	703	11	12	78.46	8.94	0.177
	C03	GSBRNA2T00097768001	BnaC03g72490D	2112	703	11	12	78.49	8.98	0.187
BnaBOR2s	A04	GSBRNA2T00093109001	BnaAnng31830D	3069	707	11	12	78.97	8.52	0.193
	C04	GSBRNA2T00156837001	BnaC04g21390D	3069	706	11	12	78.84	8.2	0.201
BnaBOR3s	A03	GSBRNA2T00111858001	BnaA03g29440D	2217	738	11	12	81.24	9.15	0.234
	C03	GSBRNA2T00125304001	BnaC03g34730D	2214	737	11	12	81.21	9.26	0.22
BnaBOR4s	A01	GSBRNA2T00083145001	BnaA02g16980D	2028	675	12	13	75.98	7.59	0.117
	A02	GSBRNA2T00069284001	BnaA02g35930D	2052	683	12	13	76.46	6.91	0.162
	C02	GSBRNA2T00063784001	BnaCnng08280D	2052	683	12	13	76.41	6.88	0.172
	C05	GSBRNA2T00068220001	BnaC05g11780D	2028	675	12	13	75.98	7.59	0.117
BnaBOR6s	A06	GSBRNA2T00147638001	BnaA06g27700D	2019	672	11	12	75.76	7.26	0.266
	C07	GSBRNA2T00133614001	BnaC07g29340D	2016	671	11	12	75.84	7.94	0.258
BnaBOR7s	A01	GSBRNA2T00121111001	BnaA01g04520D	3648	1215	23	24	134.72	7.82	0.047
	A03	GSBRNA2T00077798001	BnaA03g52100D	1983	660	9	10	74.68	8.58	0.225
	C01	GSBRNA2T00130576001	BnaC01g06010D	2013	670	11	12	75.4	7.21	0.226
	C07	GSBRNA2T00099855001	BnaC07g43840D	1986	661	9	10	74.67	8.58	0.24

### Structure and conserved motif analysis of *BnBOR* genes

**Fig. 1:**
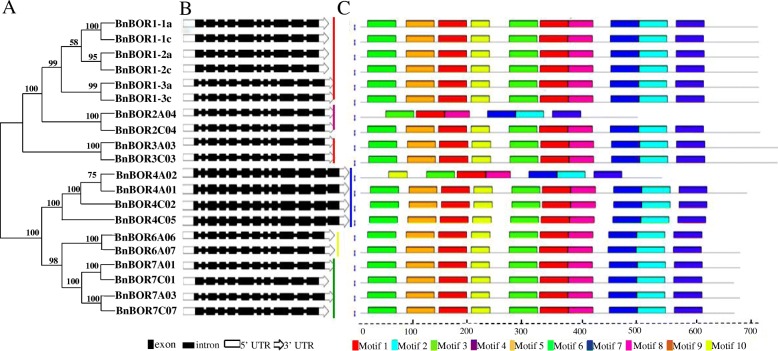
Schematic representation of the exon-intron compositions and conserved motifs in the *BnBOR* genes in *Brassica napus*. **a** Neighbour-joining (NJ) phylogenetic tree for *BnBOR*s. **b** Exon-intron organization of *BnBOR* genes. The wide black boxes represent exons, the narrow black lines represent introns, the white rectangles represent the 5’-UTR and the white arrows represent the 3’-UTR. **c** Distribution of conserved motifs in BnBOR proteins. Different motifs are shown by different colours numbered 1–10. Some motifs are highlighted with different coloured boxes with numbers. Lines represent protein regions without a detected motif

To examine the classification of the *BnBOR*s in detail, independent classification maps were constructed with the 20 *BOR* family members (Fig. 1a). According to the *Arabidopsis BOR*1–7 family members, the *BnBOR*s were divided into 6 subfamilies (Fig. 1a and Table 1). The *BnBOR1* subfamily consists of six gene members. *BnBOR2*, *BnBOR3* and *BnBOR6* have the lowest members with only two genes in each subfamily. The other two subfamilies, *BnBOR4* and *BnBOR7*, contain four members. Additionally, abiding with the classification criteria, no proteins homologous to *BOR5* were found in *B. napus*. Since intron/exon organization and numbers are typical imprints of evolution within gene families, we analysed the *BnBOR* gene structures by comparing the gDNA sequences with their corresponding coding sequences (Fig. 1b). *BnBOR1;1a*, *BnBOR1;1c (BnaC4.BOR1;1c)*, *BnBOR1;2a*, *BnBOR1;2c*, *BnBOR7A03* and *BnBOR7C07* contain 9 introns and 10 exons, while *BnBOR4A01*, *BnBOR4A02*, *BnBOR4C02* and *BnBOR4C05* consist of 12 introns and 13 exons. The other genes had 11 introns and 12 exons.

**Fig. 2:**
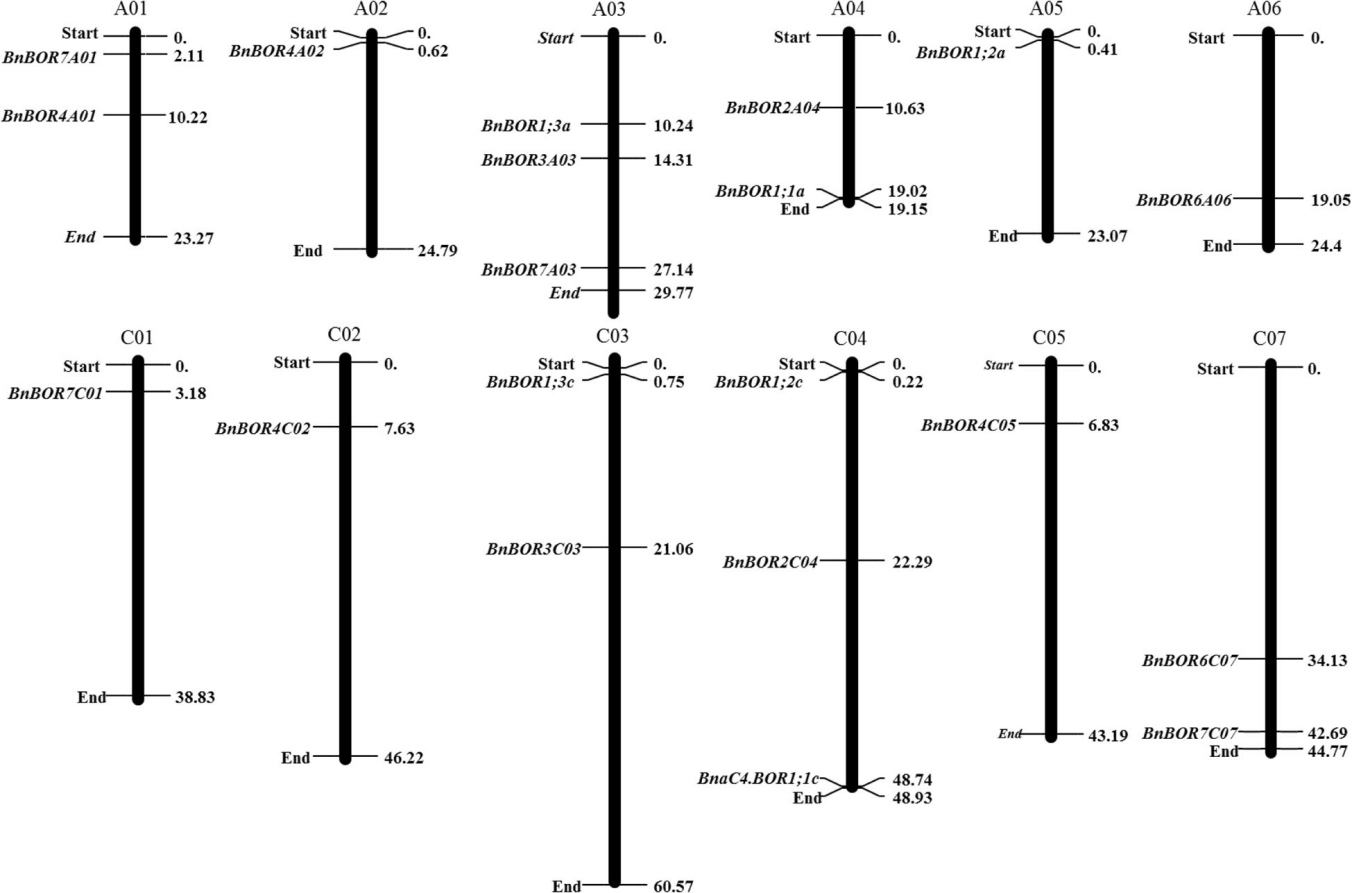
Chromosomal location of BOR members in *Brassica napus*. The gene chromosome location diagram was drawn using the MapInspect software. The size of each chromosome can be estimated from the bottom number (Mb). The 20 BnBORs are located on 12 *B. napus* chromosomes

To compare the differences in the protein structure, MEME was used to investigate the conserved motifs among the *B. napus* BOR proteins, revealing a total of 10 conserved motifs (designated motifs 1–10) (Fig. 1c). *BnBOR2A04* possesses 6 motifs, except motifs 5, 6, 9 and 10, and *BnBOR4A01* does not contain motifs 5, 6 and 9. The remaining motifs, including 1, 2, 3, 4, 7 and 8, constitute the conserved bicarbonate (HCO_3_^−^) domain, were identified by the Pfam domains and WebLogo programs, and are present in all BOR family members. The *BnBOR*s are unevenly distributed on 12 of 19 *B. napus* chromosomes (Fig. 2). The majority of *BnBOR* genes are located on chromosome arms that are associated with high rates of recombination. Ten *BnBOR* genes (*BnBOR7A01, BnBOR4A01, BnBOR4A02, BnBOR1;3a, BnBOR3A03, BnBOR7A03, BnBOR2A04, BnBOR1;1a, BnBOR1;2a,* and *BnBOR6A06*) are distributed on chromosome AA, including A01, A02, A03, A04, A05, and A06, and nine *BnBOR* genes (*BnBOR7C01, BnBOR4C02, BnBOR1;3c, BnBOR3C03, BnBOR1;2c BnBOR2C04, BnBOR1;1c, BnBOR4C05, BnBOR6C07*) are distributed on C01, C02, C03, C04, C05 and C07. Interestingly, three *BnBOR* genes (*BnBOR1;3a, BnBOR3A03a* and *BnBOR7A03*) are distributed on A03, which had an important quantitative trait locus (QTL) for B efficiency in our previous studies [34]. These results indicate that most motifs were distributed in all BOR genes, which correlates with their functional consistency and divergence.

### Phylogenetic tree for *BOR* genes in monocotyledons and dicotyledons

**Fig. 3:**
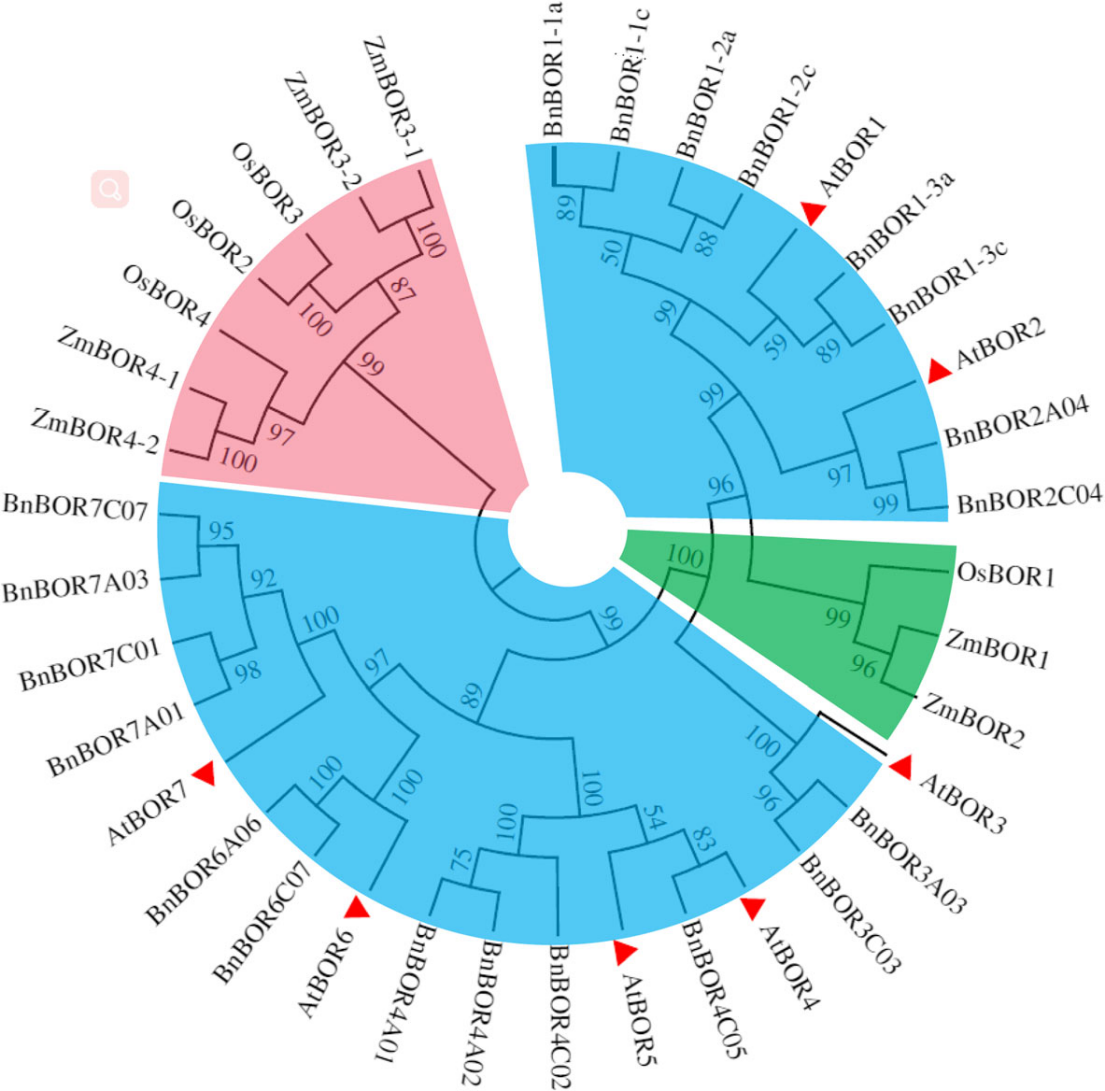
Phylogenetic tree for BORs in monocotyledons and dicotyledons. *Arabidopsis BOR* genes are highlighted with red triangles. The *BnBOR* genes are in two clades with blue colour and classified into 6 groups; *ZmBOR1* and *OsBOR1* were marked in pink and green, respectively

To examine the phylogenetic relationships among the BOR proteins in monocotyledons and dicotyledons, an unrooted phylogenetic tree was constructed from the alignment of the codon nucleotide sequences using the Neighbour-Joining method (Fig. 3). As shown in Fig. 3, the BORs could be classified into 2 main groups, which is consistent with monocotyledons and dicotyledons. Compared to monocotyledons, BnBORs from dicotyledons were more closely related to AtBORs (Fig. 3; Additional file 1: Figure S1). The phylogenetic tree and comparative sequence analysis with AtBOR1 revealed that BnBOR1s share 70–90% amino acid identity with the *Arabidopsis* B efflux transporter BOR1. Additionally, BnBOR2s are AtBOR2 orthologs, which are close to AtBOR1. Furthermore, comparative sequence analysis of the BOR1 homologue was performed with the NCBI BLAST and ClustalW2 servers. The paralogous BnBOR proteins were highly similar to each other, with an amino acid identity/similarity ranging from 71/53% to 100/99% (Additional file 1: Figure S2). These results indicate that BnBORs may function as B efflux transporter similar to AtBORs.

### Distinct *BnBOR* expression profiles in different tissues and in response to boron deficiency

**Fig. 4:**
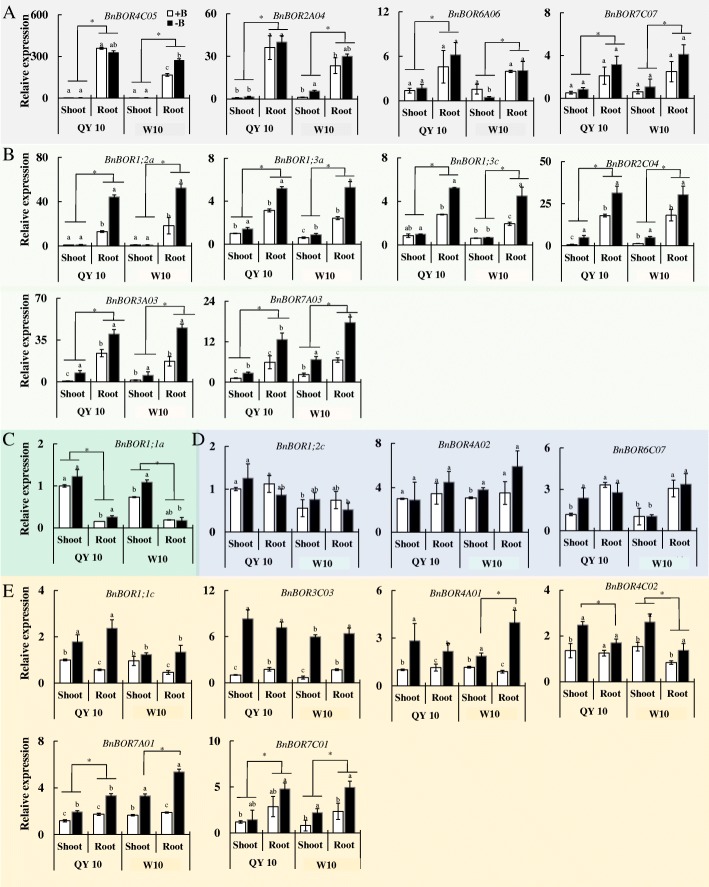
Expression profile of *BnBOR* genes in response to B stress in QY10 and W10. **a** The *BnBOR* genes that were mainly expressed in roots and were not regulated by the external B status. **b** The *BnBOR* genes that were mainly expressed in roots and were up-regulated by low B stress. **c** The *BnBOR* genes that were mainly expressed in shoots. **d** The *BnBOR* genes that were expressed in both roots and shoots and were not regulated by the external B status. **e** The *BnBOR* genes that were expressed in both roots and shoots and were up-regulated by low B stress. Different letters represent statistical significance among different B treatments and cultivars (*p* < 0.05) in the same organ. Asterisk indicates significance between shoot and root

**Fig. 5:**
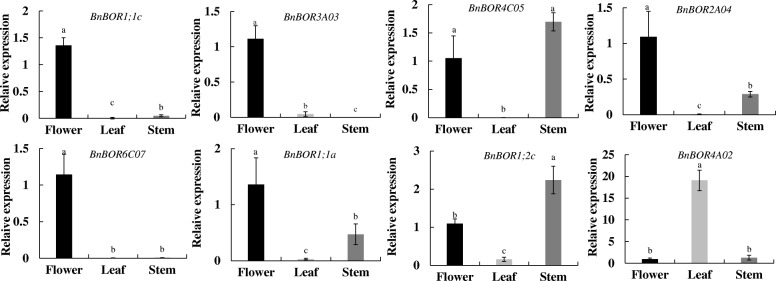
Expression profile of *BnBOR* genes in different tissues of QY 10 at the reproductive-stage

To reveal the expression profiles of BnBORs in different tissues at the seedling stage and in response to B deficiency between two cultivars, qRT-PCR was performed using gene-specific primers for 16 *BnBOR* genes. BnBOR members showed distinct expression patterns in *Brassica napus*, which could be divided into four categories based on their tissue expression and responses to B deficiency (Fig. 4). Ten genes (*BnBOR4C05, BnBOR2A04, BnBOR6A06, BnBOR7C07, BnBOR1;2a, BnBOR1;3a, BnBOR1;3c, BnBOR2C04, BnBOR3A03* and *BnBOR7A03*) displayed much higher expression in roots than shoots, especially *BnBOR4C05, BnBOR2A04* and *BnBOR1;2a* (Fig. 4a and b), and most of them were up-regulated by B deficiency (Fig. 4b), except *BnBOR4C05, BnBOR2A04, BnBOR6A06* and *BnBOR7C07* (Fig. 4a). Interestingly, we found that *BnBOR1;1a* was mainly expressed in shoots (Fig. 4c). The other *BnBOR* genes were expressed both in roots and shoots (Fig. 4d and e). Among them, the expression of *BnBOR1;2c, BnBOR4A02, and BnBOR6C07* were not affected by external B status (Fig. 4d), and the others were up-regulated by B deficiency, including *BnBOR1;1c, BnBOR3C03, BnBOR4A01, BnBOR4C02, BnBOR7A01* and *BnBOR7C01* (Fig. 4e)*.* Additionally, *BnBOR1;1c* and *BnBOR1;2c* showed higher expression levels in QY10 than in W10. Among them, the expression of *BnBOR1;1c* was up-regulated by B deficiency and the induction of *BnBOR1;1c* was more intense than in QY10. *B. napus* requires a large B supply for plant growth and reproduction, especially for the differentiation of the flowers where B is the most in demand. At reproductive stage, we found BnBOR1;1c, BnBOR3A03, BnBOR6C07 were mainly expressed in flowers and *BnBOR1;1a, BnBOR4C05, BnBOR2A04, BnBOR1;2c* showed higher expression both in flowers and stems (Fig. 5). Interestingly, the *BnBOR4A02* was distinctly expressed in leaves (Fig. 5). The diversity in the expression patterns among these genes may reveal diverse functions of B transporters in allotetraploid *B. napus.*

### Enhanced *BnBOR1;1c* expression alleviates B deficiency in shoots

**Fig. 6:**
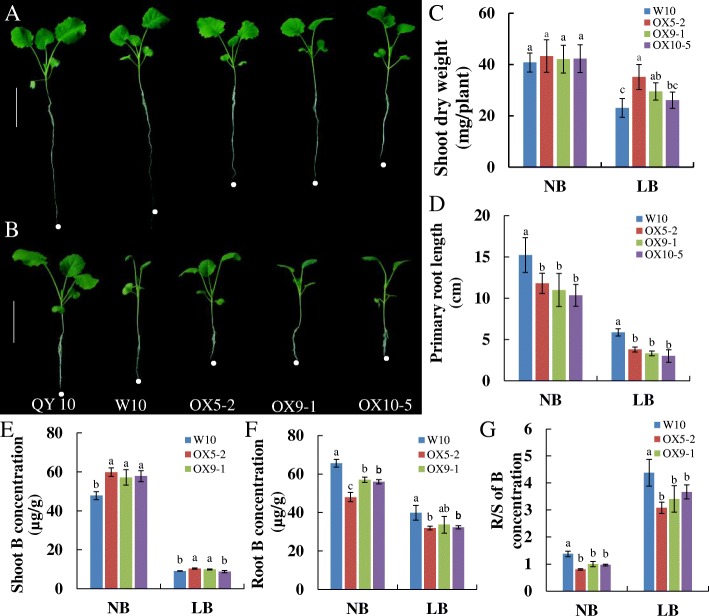
Overexpression analysis of *BnaC4.BOR1;1c* in *Brassica napus* B-inefficient W10. **a** and (**b**) Phenotypes of QY10, W10 and overexpressing lines after 10 d of growth at normal B (NB, 25 μM B) and low B (LB 0.25 μM B), respectively. **c** Shoot dry weight and (**d**) primary root length for W10 and the overexpressing lines. The (**e**) shoot, (**f**) root and (**g**) root/shoot B concentrations. Each treatment was repeated three times (*n* = 3) and error bars denote the standard deviation (SD)

We further examined whether the BOR protein BnBOR1;1c, which has a relatively high protein similarity to AtBOR1, is equally involved in the transfer of B from roots to shoots. To determine the biological function of *BnBOR1;1c* in *B. napus*, we performed a functional complementation test by inducing *35S*::*BnBOR1;1c* in the B-inefficient genotype W10. Three independent lines were used for phenotypic characterization under normal and low B conditions (Fig. 6a and b). W10 displayed a range of typical B deficiency symptoms, including stunted root and shoot growth and dark green and curved leaves under low B (Fig. 6b). The *BnaC4.BOR1;1c t*ransgenic plants (OX5–2, OX9–1 and OX10–5) displayed stronger tolerance to low B stress with significantly higher shoot dry weights than W10 under low B (Fig. 6b and c). However, the primary root length was reduced in the transgenic plants under both normal B and low B conditions (Fig. 6b and d). Additionally, the transgenic plants showed significantly higher B concentrations in shoots (Fig. 6e), but the B concentration was lower in roots relative to the wild-type W10 (Fig. 6f), which resulted in a lower R/S ratio for the B concentration (Fig. 6g). These results demonstrate that BnBOR1;1c had a similar biological function with AtBOR1, which is involved in transfer of B from roots to shoots. Importantly, enhanced *BnBOR1;1c* expression could alleviate B deficiency in shoots by improving their B concentration.

### Differential physiological responses to B deficiency between two different B-efficient cultivars

**Fig. 7:**
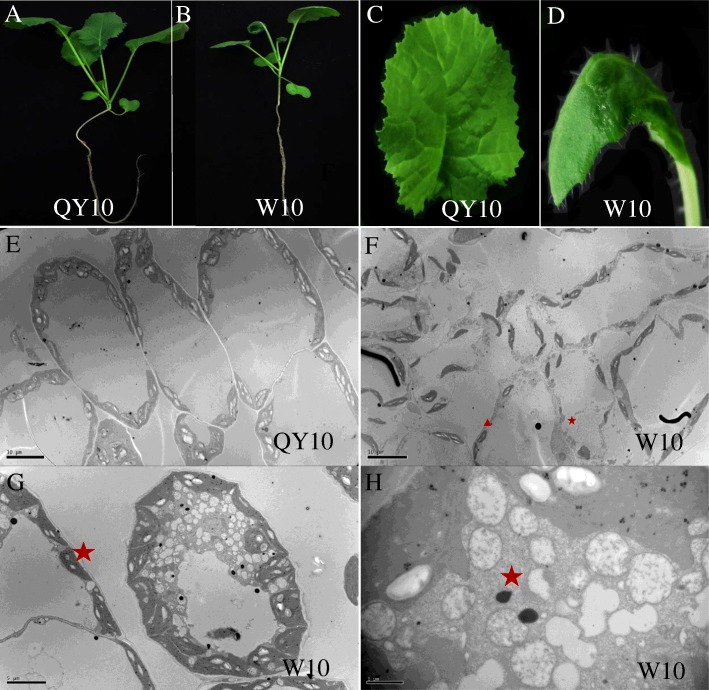
Phenotypes and transmission electron microscopy (TEM) analysis of the *Brassica napus* B-efficient cultivar QY10 and B-inefficient cultivar W10. The phenotypes of (**a**) QY10 and (**b**) W10 plants under low B (0.25 μM) conditions. The phenotypes of juvenile leaves from (**c**) QY10 and (**d**) W10. Mesophyll cells from (**e**) QY10 and (**f**-**h**) W10; red pentagrams mark the lysosomes

To evaluate the adaptation of QY10 and W10 to B deficiency, 0.25 μM and 25 μM B were used as normal and low B conditions in a hydroponic seedling culture system, respectively. Under low B conditions, the B-efficient cultivar QY10 showed better growth performance than the B-inefficient W10 (Fig. 7a and b). Moreover, W10 displayed crimped leaves and inhibited shoot apices, which were characteristic of B-deficiency symptoms (Fig. 7c and d). To examine the cellular changes underlying the morphologies of the B-deficiency symptoms in W10, we analysed the ultrastructure of the abnormal curved leaves using TEM. Mesophyll cells from QY10 were well organized and structurally intact (Fig. 7e), but the mesophyll cells from W10 appeared unordered and malformed (Fig. 7f). Additionally, the polymerization between cells became significantly weakened and the intercellular gap was enlarged (Fig. 7g). More importantly, we found a large number of lysosomes in W10 cytoplasms, which is viewed as a final destination for endocytic intracellular degradation (Fig. 7h). The lysosomes function as programmed cell death initiators and may lead to B deficiency phenotypes.

**Fig. 8:**
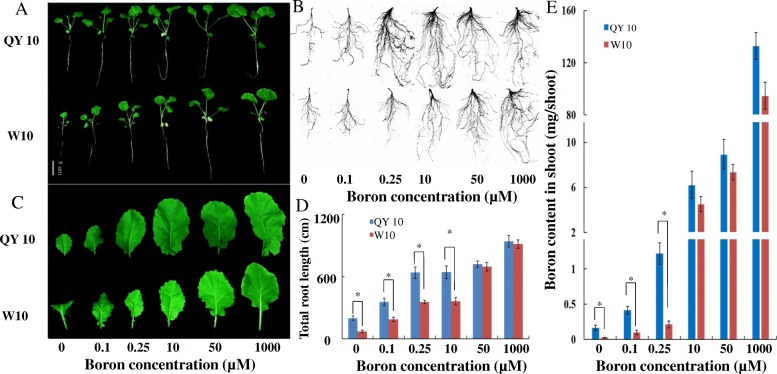
Phenotypes of the *Brassica napus* B-efficient cultivar QY10 and B-inefficient cultivar W10 in response to different B levels. **a**, **b**, and **c** The growth phenotypes of QY10 and W10 grown for 15 d at different B levels. **d** The total root length of the two cultivars. **e** The shoot B content. Each treatment was repeated three times (*n* = 3) and error bars denote the standard deviation (SD). Asterisk indicates significance between two genotypes

In the B gradient experiment, the growth of QY10 and W10 was facilitated by an increased supply of B nutrition (Fig. 8a). However, under B limitation conditions, QY10 grew significantly better than W10 until the B supply concentration was up to 10 μM, including more extended leaves and developed roots (Fig. 8a-d). Similarly, the shoot B content in QY10 was significantly higher than in W10 (Fig. 8e). When the B supply was increased to more than 10 μM, no significant differences in the B content were detected between the two cultivars. Overall, these results show that the B-efficient cultivar QY10 accumulated more B in shoots under low B stress.

### Comparison of the *BnBOR1;1c* promoter sequences between B-efficient and B-inefficient genotypes

**Fig. 9:**
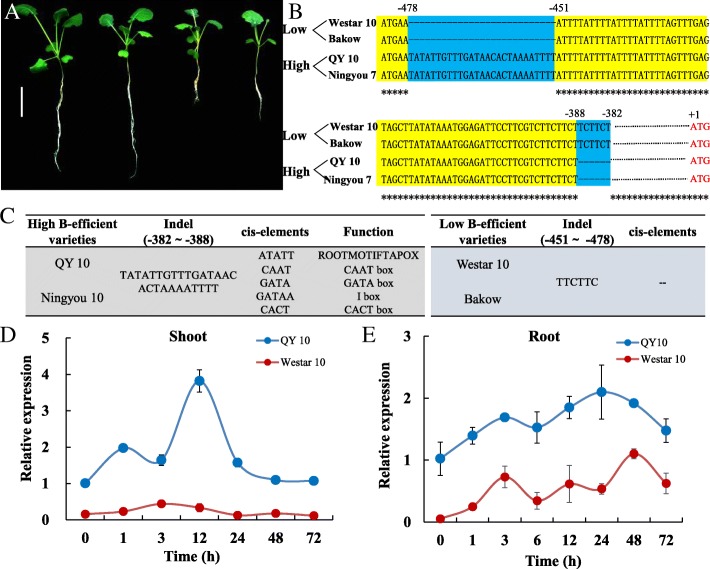
Expression and promoter analysis of *BnaC4.BOR1;1c* in B-efficient and B-inefficient cultivars. **a** Phenotypic performance of B-efficient cultivars (QY 10 and Ningyou 7) and B-inefficient cultivars (W10 and Bakow) that were cultivated in a hydroponic culture under B-limitation conditions (0.25 μM) for 10 d. **b** Sequence alignment of *BnaC4.BOR1;1c* promoters from two cultivars. **c** A 27-bp insertion in the region from − 451 bp to − 478 bp in the QY10 promoter that harbours four important cis-acting elements predicted using PLACE. Expression of *BnaC4.BOR1;1c* in (**d**) shoots and (**e**) roots from QY10 and W10; the plants were grown in 10 μM boric acid for 7 d, then transplanted in 0.25 μM B to analyse the expression after 1, 3, 12, 24, 48, and 72 h of treatment

Among the *BnBORs*, the expression of *BnBOR1;1c* was up-regulated by B deficiency and the induction of *BnBOR1;1c* was more intense in QY10 compared to W10. We next isolated and assessed the BnBOR1;1c coding sequences and promoter from the two cultivars using CNS-Genoscope. Interestingly, the coding sequence was the same and two InDels were found in the promoter regions (Additional file 1: Figure S3). One is an insertion of 27 bp at − 478 bp to − 451 bp in the QY10 promoter relative to W10. The other is an insertion of two TTC repeats at − 388 bp to − 382 bp in W10 (Fig. 9b). Interestingly, we found that the two InDels are conserved in the B-efficient genotypes Ningyou 7 and QY10, but they are lost in the B-inefficient genotypes Bakow and Westar 10 (Fig. 9c). The B-efficient and B-inefficient cultivars were identified from 210 *Brassica napus* cultivars using a B efficiency coefficient (BEC), which is the ratio of the biomass or seed yield under B deficiency to that under normal B supply [35]. Under low B conditions, Ningyou 7 and QY10 grew significantly better than Bakow and Westar 10 (Fig. 9a). Furthermore, four cis-acting elements, YACT, GATA BOX, IBOXCORE, and ROOTMOTIF TAPOX І, were identified in the 27-bp insertion regions in the QY10 promoter using the PLACE website (Fig. 9c). Among these elements, the GATA sequence is a core element in the 35CaMV promoter and ROOTMOTIF TAPOX І (ATATT) is a novel binding site for WRKY transcription factors. Quantitative RT-PCR results verified that BnaC4.BOR1;1c expression levels in the roots and shoots of QY10 plants were significantly greater than those in W10 (Fig. 9d, e). The differential expression of BnaC4.BOR1;1c may contribute to the high B efficiency in QY10 shoots. Additionally, we further analysed the cis-element in the *BnaC4.BOR1;1c* promoter in QY10 using the online software PlantCARE and found various types of cis-elements, such as stress response-, hormone response-, and development-related elements. These results indicated that the insertion in the *BnaC4.BOR1;1c* promoter of QY10 contains potential cis-acting regulatory elements, which may be responsible for the higher expression levels in QY10.

## Discussion

### BOR genes in *B. napus* and their evolution

The anion exchanger bicarbonate (HCO_3_^−^) domain related to anion transport has been widely described in bacteria, plants and animals [36]. The conserved domain in the *BOR* gene family has been reported in a number of plant species, but few have been reported in *Brassica napus,* an important oil crop cultivated worldwide. In the present study, we performed a comprehensive search for HCO_3_^−^ domain-containing BOR genes throughout the *B. napus* genome, and a total of 20 full-length *BnBOR*s were identified. *BnBOR* gene family members with high homologous conservation in their amino acid coding sequence have similar protein physicochemical parameters and structural properties, and all the members of this family are predicted to be located on the plasma membrane. In this study, these genes were divided into six distinct groups based on domain organization and phylogenetic analysis, which is highly consistent with the results from *Arabidopsis* BORs. As the *A. thaliana* genome contains 7 *BOR* genes, the number of *BOR* genes in *B. napus* is about three times that in *A. thaliana*. *B. napus* is a recent allopolyploid that originated by combining the intact genomes of *B. oleracea* and *B. rapa* and shows 20 *BOR* genes from the two immediate progenitor species *B. rapa* and *B. oleracea*. Copy number expansion of the BOR family in *B. napus* has primarily occurred through genome duplication events, and the *B. napus* genome appears to be on the more stable side. Due to the origin and evolutionary independence of the two diploids (*B. oleracea* and *B. rapa*) over the past 4.6 MYA [37], the chromosomal locations of *BnBOR*s in the A genome are not completely conserved in homologous regions in the C genome. Tandem and segmental duplication events are the main reasons for gene expansion as organisms adapt to new and changing environments [38]. The BOR genes are unevenly distributed on 12 of 19 *B. napus* chromosomes, with the majority of the *BnBOR* genes being located on chromosome arms that are associated with high rates of recombination. The uneven and clustered distribution of BOR genes has also been found in rice and maize [14, 22, 39]. The diversification of *BnBOR* genes was observed from many aspects, including phylogenesis, genomic structure, as well as chromosome rearrangement. This diversity in the structure of BOR genes is likely to be triggered by gene duplication accompanied by the loss or gain of introns and exons, which presumably contributes to protein functional diversity.

Polyploidy or whole-genome duplication (WGD) plays key roles in the evolution of gene families throughout the evolutionary history of plants, and helps plants adapt to new and changing environments [38]. Divergent expression patterns in different tissue responses to B deficiency were detected among BnBOR paralogs (Fig. 4), indicating the differential roles of each member in the regulation of B nutrition in *B. napus*. *BnBOR4C05, BnBOR2A04* and *BnBOR1;2a* showed root-specific expression (Fig. 4a and b) and *BnBOR1;1a was* mainly expressed in shoots (Fig. 4c). Additionally, *BnBOR3C03* was strongly induced by B deficiency (Fig. 4e). B requirement for the differentiation of the flowers is much higher than for vegetative growth. In our previous study, we found knockdown of *BnaC4.BOR1;1c* caused severe inhibition of inflorescence growth only under boron limitation [19]. In this research, we found other *BnBORs* also showed a higher expression in flowers (Fig. 5), which may exist functional redundancy for inflorescence development by facilitating boron transport to the growing reproductive tissues. All these results reveal a more complex network of B nutrition in *B. napus* with complex genomes.

### Genetic effects of *BnBOR1;1c* on B efficiency

B-deficiency-induced alterations in gene expression profiles and proteomic profile in *C. sinensis* and *Brassica napus* [34, 40–42]*.* The function of the *BOR1* gene has been well-characterized in *Arabidopsis*, rice, maize and wheat [10, 14]. However, the genetic basis underlying B efficiency in *Brassica napus* remains poorly elucidated. The allotetraploid rapeseed is extremely sensitive to B deficiency and shows a notable reduction in seed yield and quality with a low B supply [10, 14, 43]. Based on the comparative differential physiological responses to B deficiency between genotypes, we found that the B-efficient cultivar QY10 appears better growth performance (Fig. 7) and accumulates more B in shoots under low B stress (Fig. 8). We examined transgenic rapeseed oil by inducing *35S*::*BnBOR1;1c* in the B-inefficient genotype W10. The transgenic plants showed better growth and higher B contents in shoots under B deficiency than in wild type W10 (Fig. 6), indicating that *BnBOR1;1c* may function in B transport, similar to *AtBOR1*, while *BnBOR1;1c* overexpression conferred a strong tolerance to low B stress in *B. napus*.

We also found that *BnBOR1;1c* expression was much higher in both roots and shoots in the B-efficient cultivar QY10 (Fig. 6 and Fig. 9d and e). Interestingly, the coding sequence for *BnaC4.BOR1;1c* was the same as in QY10 and the B-inefficient cultivar W10, though two InDels were found in the promoter regions between them (Fig. 9b). Furthermore, we identified four significant *cis*-acting elements among the 27-bp InDels in QY10, including YACT, GATA box, I box, and ROOTMOTIF TAPOX І. These elements are related to mesophyll expression and activating reporter gene expression and involved in light-responsive and root-specific elements. Gowik [44] reported that the YACT motif is a key component in Mem1 (mesophyll expression module 1) found in the promoter of the phosphoenolpyruvate carboxylase (ppcA1) in the C4 plant *Flaveria trinervia*. The ROOTMOTIF TAPOX was identified for the first time in the *rol*D promoter in *Agrobacterium rhizogenes* and has been proven to be responsible for the expression of the *gus* gene in the roots of transgenic tobacco plants [45]. In fact, the cis-elements present upstream of the target genes can regulate gene expression and alter gene function. Ye et al. reported that an InDel in the Sl-*ALMT9* promoter contributes to fruit malate accumulation and enhances aluminium tolerance in tomato [46]. Additionally, InDel analysis in other *B. napus* genotypes revealed that the 27-bp InDels are conserved between B-efficient (QY10 and Ningyou 7) and B-inefficient (Westar 10 and Bakow) types (Fig. 9b and c). Thus, these InDels can be used for molecular marker-assisted selection of *B. napus* to improve plant B nutrition. However, the biological roles of these InDels in the *BnBOR1;1c* promoter require further study.

## Conclusions

In conclusion, 20 *BnBOR* genes were identified in the *Brassica napus* genome and named according to their phylogenetic relationships. The diversity of expression patterns among these genes may reveal diverse functions in allotetraploid *B. napus.* By investigating the differential physiological responses to B deficiency, the B-efficient cultivar QY10 showed better growth performance and a higher dry weight and B concentration than the B-inefficient cultivar W10 under B limitation. Among the 20 identified *BnBOR* genes, the expression of *BnaC4.BOR1;1c* was much higher in both the roots and shoots of QY10. Moreover, two InDels were found in the promoter regions of the two cultivars, and these InDels are conserved between the B-efficient and B-inefficient genotypes. Importantly, overexpressing *BnaC4.BOR1;1c* could alleviate B-deficiency in the B-inefficient cultivar W 10. All these results provide comprehensive insights into the BOR family members in *B. napus* and highlight the diversity of the family members, which could be valuable for further biological function studies focused on *BnBOR* genes*.*

## Additional file

Additional file 1: **Figure S1.** Phylogenetic tree for BORs in *Brassica napus*. The BOR phylogenetic tree was generated by MEGA 6.0 with the Neighbour-joining (NJ) method and 1000 replicates bootstraps and based on the amino acid sequences of the 20 BnBOR genes and 7 AtBOR genes. The AtBORs are marked by red diamonds. **Figure S2.** Identity/similarity matrix for the BnBOR proteins. Amino acid identity and similarity are indicated by the first and second number. **Figure S3.** BLAST analysis of the promoter region from *BnaC4.BOR1;1c* in different *Brassica napus* genotypes. **Table S1.** Sequences of the primers used for PCR. **Table S2.** Putative cis-elements in the *BnaC4.BOR1;1c* promoter region in *B. napus.* (DOCX 219 kb)

## Abbreviations

B: Boron
BEC: B efficiency coefficient
*BnBOR1s*: Boron transporter genes in *Brassica napus*
*q*RT-PCR: Quantitative real-time PCR
QTL: quantitative trait locus
QY10: Qingyou 10
RG-II: pectic polysaccharide rhamnogalacturonan II
TEM: Transmission Electron Microscopy
W10: Westar 10
WGD: whole-genome duplication
