# Supplementary material for: Molecular characterization of the genome-wide BOR transporter gene family and genetic analysis of BnaC04.BOR1;1c in Brassica napus
Source: BMC Plant Biol. 2018 Sep 14;18:193. doi: 10.1186/s12870-018-1407-1 (PMC6137915; doi:10.1186/s12870-018-1407-1)
Supplement: Supplementary file 1 — Figure S1. Phylogenetic tree for BORs in Brassica napus. The BOR phylogenetic tree was generated by MEGA 6.0 with the Neighbour-joining (NJ) method and 1000 replicates bootstraps and based on the amino acid sequences of the 20 BnBOR genes and 7 AtBOR genes. The AtBORs are marked by red diamonds. Figure S2. Identity/similarity matrix for the BnBOR proteins. Amino acid identity and similarity are indicated by the first and second number. Figure S3. BLAST analysis of the promoter region from BnaC4.BOR1;1c in different Brassica napus genotypes. Table S1. Sequences of the primers used for PCR. Table S2. Putative cis-elements in the BnaC4.BOR1;1c promoter region in B. napus. (DOCX 219 kb) [file 12870_2018_1407_MOESM1_ESM.docx]

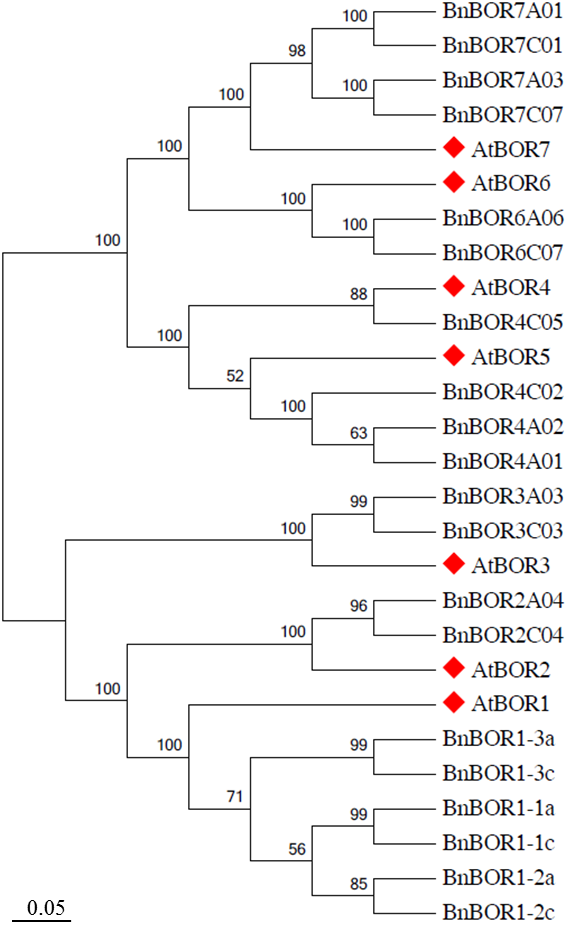


**Additional file 1: Figure S1** Phylogenetic tree of BORs in *Brassica napus*. The BORs phylogenetic tree was generated by MEGA 6.0 with Neighbor joining (NJ) and 1,000 replicates bootstraps, based on the amino acid sequences of 20 BnBOR genes and 7 AtBOR genes. The AtBORs were marked with red diamond.

**Figure S2** Identity ⁄ similarity matrix of BnBORs proteins. Amino acid identity and similarity are indicated by the first and second number.

**Figure S3** BLAST the promoter region of *BnaC4.BOR1;1c* in different *Brassica napus* genotypes.

**Table S1** Sequences of the primers used for PCR

| **Gene** | **Primer** | **Gene** | **Primer** |
| --- | --- | --- | --- |
| *BnBOR1;1c* (cDNA) | F-5'-ATGGAAGAGACCTTTGTGCCGTTTGAAG | BnBOR4C05 | F-5'-AAGCGGTGATGAAGAGCAAC |
|  | R-5'-TCCCTTGAACCAGTCCTCGTTCAACTGA |  | R-5'-TAAGCGAAGTATCCCCATAGAACT |
| *BnBOR1;1c* | F-5'-CCTTTAATCAATCCCCACTATCAAAGATTTG | BnBOR4A01 | F-5'-AGCAGACTATGGAGTTCCTCTG |
|  | R-5'-GCCAAGAAGAGGTCTCGTCCAAGTTCAGAC |  | R-5'-CCGCAAAAATATAGGCTGGG |
| *BnBOR1;1a* | F-5'-ACCAATTCTGGGAGAGAATCC | BnBOR4A02 | F-5'-ATTCGTGGAGAAAGTTCCGTAT |
|  | R-5'-CCTGCGATTGGGATCCAC |  | R-5'-CTCGTACTCAGAGGCATCG |
| BnBOR1;2a | F-5'-GTTTAAAAGAACTGAAAGAATCAACG | BnBOR4C02 | F-5'-GTTCGTGGAGAAAGTTCCTTAC |
|  | R-5'-CAGCAACGCATCCTCCC |  | R-5'-CCTCGTACTCAGAGGCATCT |
| BnBOR1;2c | F-5'-CATCACATGCAGACACCATTG | BnBOR6A06 | F-5'-TTCTCGTTAGGTCTGTTAATCACT |
|  | R-5'-CTACCATTGTGGACTGAAGGAG |  | R-5'-CCTTGATTACAGTCCAATGATGC |
| BnBOR1;3a | F-5'-ACTTCGTATCCAGGAGACTCG | BnBOR6C07 | F-5'-CTCGTTAGGTCTGTTGATCACA |
|  | R-5'-CTAAACACTTGAGACAAACTCCTAC |  | R-5'-TCCTTGATTACAGTCCAATGGTAT |
| BnBOR1;3c | F-5'-TGCAACGTTCACTATTTTCCAG | BnBOR7A01 | F-5'-AAATCCCTTTTGAGGACTATGGT |
|  | R-5'-GCTGCGAGATTGAAGGGTAAC |  | R-5'-TGAACTCATTGCCACCATAATAC |
| BnBOR2C04 | F-5'-CGAGACATTCACGGGAAATCTT | BnBOR7A03 | F-5'-CCAGCTTATCACTATGACATCTTTATTC |
|  | R-5'-CAACACATCCTCCAACCATCAC |  | R-5'-GCCAATGCCTTGGGTGATT |
| BnBOR2A04 | F-5'-CGACGACATTCACAGGGAATCTA | BnBOR7C01 | F-5'-CGGCGAGCAGTTAAGCC |
|  | R-5'-GCAACACATCCTCCAACCATTAA |  | R-5'-CTGAAGCAGTGAAGGTATGTG |
| BnBOR3A03 | F-5'-ATGTGCCTGTTGTTTACATTATC | BnBOR7C07 | F-5'-GTGTGTGTCTGGACAGCT |
|  | R-5'-GATGACACCATTAGATGGAGGTATA |  | R-5'-CCAGATTTTGGTACAAGAAACTCC |
| BnBOR3C03 | F-5'-ATGTGCCTGTAGTTTACATTGTT | *Actin* (*Brassica napus*) | F-5'-ACAGTGTCTGGATCGGTGGTTC |
|  | R-5'-CATTAGATGGAGGGATCCCAAT |  | R-5'-TGCCTCATCATACTCAGCCTTG |

**Table S2** Putative cis-elements in the promoter region of *BnaC4.BOR1;1c* in *B. napus*
